# Bovine Kobuvirus in Europe

**DOI:** 10.3201/eid1505.081427

**Published:** 2009-05

**Authors:** Gábor Reuter, László Egyed

**Affiliations:** ÁNTSZ Regional Institute of State Public Health Service, Pécs, Hungary (G. Reuter); Veterinary Medical Research Institute of the Hungarian Academy of Sciences, Budapest, Hungary (L. Egyed)

**Keywords:** Viruses, kobuvirus, cattle, picornavirus, diarrhea, bovine, Europe, letter

**To the Editor**: Picornaviruses (family *Picornaviridae*) are small, nonenveloped viruses with single-stranded, positive-sense genomic RNA. Picornaviruses are currently divided into 8 genera: *Enterovirus, Aphthovirus, Cardiovirus, Hepatovirus, Parechovirus, Erbovirus, Teschovirus,* and *Kobuvirus* (*1*). To date, the genus *Kobuvirus* consists of 2 officially recognized species, *Aichi virus* and *Bovine kobuvirus,* and 1 porcine kobuvirus as a candidate species (*2*–*4*). Aichi virus (strain A846/88) was first isolated in 1991 from feces of a person with acute gastroenteritis (*2*). Bovine kobuvirus (strain U-1) was detected in 2003 in bovine serum and fecal samples from clinically healthy cattle (*3*); in 2008, it was isolated from cattle with diarrhea (*5*). Aichi virus and bovine kobuvirus were first isolated in Japan. Porcine kobuvirus (strain S-1-HUN) was recently identified from domestic pigs in Hungary (*4*). Aichi viruses have been also detected in other countries in Asia (*6*), Europe (*7*,*8*), South America (*7*), and northern Africa (*9*). Bovine kobuvirus, however, has not been detected outside Asia (Japan and Thailand) (*3*,*5*).

Kobuvirus genomes are ≈8.2–8.4 kb and have a typical picornavirus genome organization, including leader (L) protein following structural (VP0, VP3, and VP1) and nonstructural (2A–2C and 3A–3D) regions (*1*,*3*,*4*). The genetic identity on coding regions of Aichi virus, bovine kobuvirus strain U-1, and porcine kobuvirus strain S-1-HUN is between 35% (L protein) and 74% (3D region) (*3*,*4*). We report the detection of bovine kobuvirus in Europe.

In February 2002, a total of 32 fecal samples were collected from cattle (*Bos taurus*) in a closed herd of 870 animals in central Hungary; age groups were 1–9 days (n = 6), 14–17 days (n = 4), 6–7 months (n = 5), and 1–7.6 years (n = 17). In February 2008, 26 more samples were collected from animals <20 days of age on this farm. On the sampling days, no diarrhea was reported.

Reverse transcription–PCR was performed by using a new generic kobuvirus primer (UNIV-kobu-F, forward, 5′-TGGAYTACAAG(/R)TGTTTTGATGC-3′, corresponding to nucleotides 7491–7512 of strain U-1 and UNIV-kobu-R, reverse, 5′-ATGTTGTTRATGATGGTGTTGA-3′, corresponding to nucleotides 7686–7707 of strain U-1). The primer design was based on the viral sequences of the Aichi virus (AB040749), bovine kobuvirus strain U-1 (bovine, AB084788), and bovine kobuvirus strain S-1-HUN (porcine, EU787450), which amplify a 216-nt region of 3D (RNA-dependent RNA polymerase region) of all species. The continuous 862-nt 3D and 3′ untranslated region (UTR) of the genome was determined by using 5′/3′RACE Kit (2nd Generation; Roche Diagnostics GmbH, Mannheim, Germany) and primers UNIV-kobu-F and Z20-F-7729 (5′-CCAACATCCTGACTTCTCTCCT-3′, corresponding to nucleotides 7729–7750 of strain U-1). PCR products were sequenced directly in both directions by using the BigDye Reaction Kit (Applied Biosystems, Warrington, UK), the PCR primers, and an automated sequencer (ABI PRISM 310 Genetic Analyzer; Applied Biosystems, Stafford, TX, USA). Phylogenetic analysis was conducted by using MEGA version 4.1 (*10*). The sequence of this bovine kobuvirus strain (kobuvirus/bovine/Aba-Z20/2002/Hungary) was submitted to GenBank under accession no. FJ225406.

Of the 32 samples collected in 2002, two (6.25%), from 1-year-old animals, were positive for bovine kobuvirus; however, no kobuvirus was found in the samples from 2008. The 2 partial 3D regions (216 nt) were genetically identical. Strain kobuvirus/bovine/Aba-Z20/2002/Hungary (FJ225406) had 89%–94% nucleotide and 96%–100% amino acid identities to the 19 known Asian bovine kobuvirus strains in GenBank. Strain Z20 had 93% and 95% nucleotide identities to U-1 in 3D/3′-UTR (862 nt) and 3′-UTR (174 nt) regions, respectively. Phylogenetic analysis of the overlapping partial 3D nucleotide sequences of bovine kobuvirus strain Z20 from Hungary, together with all published bovine kobuvirus strains available in the GenBank database, are shown in the Figure. Aichi virus and porcine kobuvirus were included in the tree as outlier viruses. The phylogenetic tree confirmed that strain Z20 belonged to bovine kobuviruses (Figure).

**Figure:**
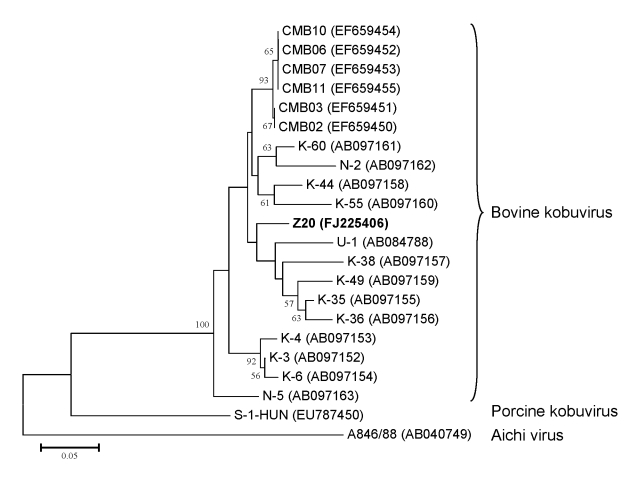
Phylogenetic tree of bovine kobuvirus (kobuvirus/bovine/Aba-Z20/2002/Hungary, in **boldface**) based on the 455-nt fragment of the kobuvirus 3D regions. The phylogenetic tree was constructed by using the neighbor-joining clustering method with distance calculation and the maximum composite likelihood correction for evolutionary rate with help of MEGA version 4.1 software (*10*). Bootstrap values (based on 1,000 replicates) are given for each node if >50%. Reference strains were obtained from GenBank. Scale bar indicates nucleotide substitutions per site.

Our detection of bovine kobuviruses in Europe confirms a wider geographic presence of this type of picornavirus in cattle and suggests that bovine kobuvirus is common and potentially distributed worldwide. Genetic diversity was seen, based on the 3D regions of bovine kobuviruses; however, this region shows the highest genetic identity among the kobuvirus genetic regions (*3*,*4*). Strain Z20 also confirms the 174-nt 3′-UTR region of bovine kobuvirus. At this time it is not clear what diseases (including gastroenteritis) are associated with bovine kobuvirus (*3*,*5*). In addition to the bovine kobuvirus, 2 other RNA viruses that are transmitted by the fecal–oral route (genotypes GIII/1 and GIII/2 of bovine noroviruses and rotavirus) were detected at the same time from these apparently healthy animals. More epidemiologic and molecular studies are required to determine the relevance, distribution, and diversity of bovine kobuvirus in cattle.

## References

[1] International Committee on Taxonomy of Viruses. Virus taxonomy 2008 [cited 2009 Feb 23]. Available from http://www.ictvonline.org/virusTaxonomy.asp?version=2008

[2] Yamashita T, Kobayashi S, Sakae K, Nakata S, Chiba S, Ishihara Y, Isolation of cytopathic small round viruses with BS-C-1 cells from patients with gastroenteritis. J Infect Dis. 1991;164:954–7. 1658159 10.1093/infdis/164.5.954

[3] Yamashita T, Ito M, Kabashima Y, Tsuzuki H, Fujiura A, Sakae K. Isolation and characterization of a new species of kobuvirus associated with cattle. J Gen Virol. 2003;84:3069–77. 10.1099/vir.0.19266-0 14573811

[4] Reuter G, Boldizsár Á, Kiss I, Pankovics P. Candidate new species of *Kobuvirus* in porcine hosts. Emerg Infect Dis. 2008;14:1968–70. 10.3201/eid1412.080797 19046542 PMC2634637

[5] Khamrin P, Maneekarn N, Peerakome S, Okitsu S, Mizuguchi M, Ushijama H. Bovine kobuviruses from cattle with diarrhea. Emerg Infect Dis. 2008;14: 985–6. 10.3201/eid1406.070784 18507924 PMC2600271

[6] Pham NT, Khamrin P, Nguyen TA, Kanti DS, Phan TG, Okitsu S, Isolation and molecular characterization of Aichi viruses from fecal specimens collected in Japan, Bangladesh, Thailand, and Vietnam. J Clin Microbiol. 2007;45:2287–8. 10.1128/JCM.00525-07 17522267 PMC1932998

[7] Oh DY, Silva PA, Hauroeder B, Deidrich S, Cardoso DD, Schreier E. Molecular characterization of the first Aichi viruses isolated in Europe and in South America. Arch Virol. 2006;151:1199–206. 10.1007/s00705-005-0706-7 16421634

[8] Ambert-Balay K, Lorrot M, Bon F, Giraudon H, Kaplon J, Wolfer M, Prevalence and genetic diversity of Aichi virus strains in stool samples from community and hospitalized patients. J Clin Microbiol. 2008;46:1252–8. 10.1128/JCM.02140-07 18256215 PMC2292896

[9] Sdiri-Loulizi K, Gharbi-Khélifi H, de Rougemont A, Chouchane S, Sakly N, Ambert-Balay K, Acute infantile gastroenteritis associated with human enteric viruses in Tunisia. J Clin Microbiol. 2008;46:1349–55. 10.1128/JCM.02438-07 18287312 PMC2292941

[10] Tamura K, Dudley J, Nei M, Kumar S. MEGA4: Molecular evolutionary genetics analysis (MEGA) software version 4.0. Mol Biol Evol. 2007;24:1596–9. 10.1093/molbev/msm092 17488738

